# Congenital Duodenal Stenosis Presenting with Bowel Obstruction Caused by Food Impaction in a Three-Year-Old Boy: A Case Report

**DOI:** 10.70352/scrj.cr.26-0165

**Published:** 2026-06-18

**Authors:** Koki Higashi, Yuhki Koike, Yuki Sato, Yuka Nagano, Tadanobu Shimura, Takahito Kitajima, Kohei Matsushita, Yoshinaga Okugawa, Yuji Toiyama

**Affiliations:** 1 Department of Gastrointestinal and Pediatric Surgery, Mie University Graduate School of Medicine, Tsu, Mie, Japan; 2 Department of Genomic Medicine, Mie University Graduate School of Medicine, Tsu, Mie, Japan

**Keywords:** congenital duodenal stenosis, food impaction, trisomy 21, bowel obstruction

## Abstract

**INTRODUCTION:**

Congenital duodenal stenosis (CDS) is often associated with trisomy 21 and is typically diagnosed in the neonatal period. However, some cases may present beyond infancy with episodes of acute gastroenteritis or foreign body impaction, which makes diagnosis challenging. This report describes a case in which a young child with trisomy 21 was diagnosed with CDS following an episode of acute bowel obstruction caused by an impacted mushroom.

**CASE PRESENTATION:**

A male infant was born at term without abnormal prenatal findings, including polyhydramnios. Although he was diagnosed with trisomy 21 after birth, no obvious congenital anomalies were identified. He began complementary feeding at 6 months of age and transitioned to a regular toddler diet at 2 years of age. At 3 years of age, he presented with recurrent vomiting at a local hospital and was initially treated for acute gastroenteritis. An upper gastrointestinal contrast study and contrast-enhanced CT raised suspicion for impaired passage at the duodenum. Consequently, the patient was referred to our department for further evaluation and management. Careful review of the CT images obtained at the referring hospital revealed a mushroom-shaped foreign body in the descending portion of the duodenum. A repeat contrast study demonstrated not only the foreign body but also indicated an underlying duodenal stenosis at the same site. Upper gastrointestinal endoscopy confirmed the duodenal stenosis and revealed an impacted mushroom blocking the opening, which was successfully removed endoscopically. Laparotomy was performed via an inverted Y-shaped umbilical incision, and a membranous stenosis in the descending portion of the duodenum was identified. Membrane resection and duodenoplasty were performed. The patient’s postoperative course was favorable, and no recurrence has been observed during follow-up.

**CONCLUSIONS:**

Late-onset CDS can be detected following food impaction. This case highlights the importance of comprehensive imaging in such cases, including a contrast study and CT, which facilitates both endoscopic removal of the impacted food and definitive surgical management of the duodenal stenosis. Clinicians should maintain a high index of suspicion for CDS in children presenting with sudden or recurrent vomiting at any age, particularly in those with trisomy 21.

## Abbreviations

CDO
: congenital duodenal obstruction
CDS
: congenital duodenal stenosis

## INTRODUCTION

Congenital duodenal atresia and CDS are relatively rare, with an estimated incidence of 1 in 6000–10000 live births.^1)^ CDO as a result of congenital duodenal atresia or CDS is associated with trisomy 21 (Down syndrome) in around a third of cases.^2)^ In CDO, polyhydramnios is frequently observed during the fetal period, and affected infants often present with recurrent vomiting from birth; therefore, most cases are diagnosed in the neonatal period. However, several reports have described patients diagnosed and treated after infancy, with some cases identified in adulthood.^3–5)^ This report describes a case in which a 3-year-old boy with trisomy 21 developed intestinal obstruction as a result of an impacted mushroom, which led to the diagnosis of CDS.

## CASE PRESENTATION

**Fig. 1:**
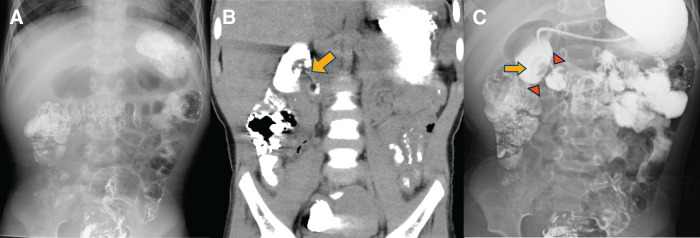
(**A**) Abdominal X-ray at admission to our institution showed an abnormal gastric bubble and duodenal bulb dilation, suggestive of upper gastrointestinal tract stenosis. (**B**) Abdominal CT image showing a mushroom-shaped foreign body in the descending portion of the duodenum (arrow). (**C**) An upper gastrointestinal contrast image showing a hemispherical foreign body in the descending portion of the duodenum (arrow) and a contrast filling defect suggesting the stenosis (arrowheads) at the same site. Contrast medium passed smoothly beyond the descending portion of the duodenum, with no evidence of obstruction in the distal segment.

**Fig. 2:**
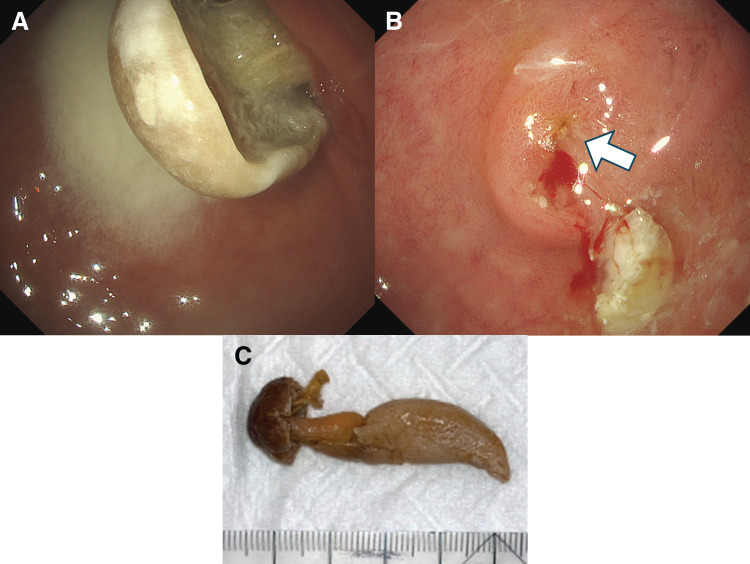
Gastrointestinal endoscopic and photographic images of the impacted mushroom that was endoscopically removed. (**A**) The mushroom is impacted at the site of the membranous stenosis in the descending portion of the duodenum. (**B**) Membranous stenosis with a pinhole-like orifice was observed (arrow) after removal of the mushroom. (**C**) Photograph of the extracted mushroom, which measured 47 mm in length with a stem diameter of 10 mm and a cap diameter of 15 mm.

A male infant was born at 37 weeks and 4 days of gestation via spontaneous vaginal delivery with a birth weight of 2718 g. There were no abnormal prenatal findings, including polyhydramnios. He was diagnosed with trisomy 21 after birth; however, no obvious congenital anomalies associated with chromosomal abnormalities were identified. He began complementary feeding at around 6 months of age. Although he experienced occasional episodes of vomiting after relatively large meals, he was managed with observation in view of his age and comorbid condition, and his symptoms subsequently resolved spontaneously. He transitioned to a regular toddler diet at around 2 years of age, with no recurrence of symptoms. At 3 years of age, he was admitted to a local hospital with recurrent vomiting. Acute gastroenteritis was suspected, and conservative management based on fasting was initiated. However, there was little improvement in his symptoms. The findings on an upper gastrointestinal contrast study and contrast-enhanced CT raised suspicion for impaired passage at the duodenum. Therefore, the patient was referred to our department for further evaluation and management. A supine abdominal X-ray at admission to our institution showed an abnormal gastric bubble and duodenal bulb dilation, suggestive of upper gastrointestinal tract stenosis (**Fig. 1A**). CT imaging at the referring hospital suggested obstruction of passage in the descending portion of the duodenum, along with a mushroom-shaped foreign body at the same site (**Fig. 1B**). To reassess the presence of a foreign body and other possible causes of obstruction, a repeat upper gastrointestinal contrast study was performed. The study revealed contrast retention, a foreign body, and membranous stenosis in the descending portion of the duodenum (**Fig. 1C**). An upper gastrointestinal endoscopy performed on hospital day 2 further confirmed this, revealing duodenal stenosis in the descending portion of the duodenum with an impacted mushroom (**Fig. 2**). After endoscopic removal of the foreign body, a repeat contrast study again suggested the presence of a membranous stenosis at the same location. Therefore, definitive surgery was performed on hospital day 4. Laparotomy was performed via an inverted Y-shaped umbilical incision, and a caliber change was observed in the descending portion of the duodenum. After a longitudinal incision of the duodenal wall and inspection of the lumen, a membrane with an orifice was identified in the same area as the caliber change, and the duodenal web was found to be preampullary. We diagnosed duodenal membranous stenosis and performed duodenoplasty with membrane excision using transverse closure. We also performed an inverted appendectomy in view of the presence of a mobile cecum. The postoperative course was uneventful, with no recurrence of vomiting. He resumed oral intake without the need for special nutritional support and was able to tolerate a regular diet before discharge. He was discharged on POD 8. His symptoms have not recurred during follow-up, and his clinical course remains favorable.

## DISCUSSION

We encountered a case in which a small child with trisomy 21 developed intestinal obstruction because of CDS complicated by food impaction. Detailed preoperative imaging evaluation allowed an accurate diagnosis and facilitated safe surgical management of the CDS. Although CDS is typically diagnosed during the neonatal period, a small proportion of cases present beyond infancy. In these late-presenting cases, the previously undiagnosed duodenal stenosis was identified at the time of presentation with acute gastroenteritis or foreign body impaction.^6,7)^ Most reported cases of late-onset CDS are due to intrinsic causes, with membranous stenosis accounting for almost all cases, whereas a minority are attributed to extrinsic causes such as annular pancreas.^5,7,8)^ Similar to neonatal cases, the stenotic lesion is most frequently located near the ampulla of Vater, with few reports of stenosis at other sites.^8–11)^ Based on these findings, the etiology and anatomical location of the stenosis may not play a significant role in the development of late-onset CDS. Meanwhile, an inverse correlation between the degree of luminal narrowing and the age at symptom onset has been reported, indicating that the timing of presentation varies according to the extent of stenosis.^12)^ The transition to solid foods during weaning may also contribute to the onset of symptoms in children with CDS at around 1 year of age, and symptoms of CDS can be masked or underestimated in children with Down syndrome because of developmental delays.^13)^ Therefore, careful evaluation for this condition is warranted even in older children with Down syndrome. In the present case, symptoms developed at 3 years of age following food impaction. Although our patient was older than the typical age of presentation, the coexistence of trisomy 21 may have contributed to the delayed recognition of CDS.

Late-onset CDS can be challenging to diagnose, given that plain abdominal radiographs frequently lack the typical double-bubble sign and reduced bowel gas in the distal bowel, potentially contributing to delayed diagnosis.^14)^ Although upper gastrointestinal contrast studies are often required for a definitive diagnosis, CT imaging may clearly delineate the location of the stenotic segment.^15)^ Identification of a coexisting foreign body impaction may be even more challenging than establishing the diagnosis of CDS. As observed in the present case, CDS can be diagnosed following food impaction, with causative foods reported to include mushrooms, nuts, and fruit seeds.^7,13,16)^ Consumption of edible mushrooms varies geographically, but is particularly prevalent in East Asia. Several cases of mushroom-induced food impaction have been reported in Japan.^7,17,18)^ In these cases, food impaction was not easily recognized and was sometimes detected incidentally during endoscopic assessment for CDS or at the time of surgery. In our patient, the coexistence of trisomy 21 and the CT findings at the referring hospital raised suspicion for duodenal stenosis complicated by a foreign body. This suspicion was confirmed by an upper gastrointestinal contrast study performed at our institution. Accordingly, we were able to proceed with endoscopic retrieval of the foreign body, make a definitive diagnosis of CDS, and treat the stenosis surgically.

Surgical management of CDS that presents late is similar to that in neonates and typically involves duodenoplasty with membrane excision or bypass of the obstruction with duodenoduodenostomy. Laparoscopic surgery has been shown to result in earlier resumption of feeding and a shorter hospital stay in comparison with open surgery; however, long-term outcomes are reported to be comparable between the 2 approaches.^19)^ Another study demonstrated that the coexistence of trisomy 21 does not adversely affect the surgical outcomes of CDS.^10)^ Furthermore, endoscopic interventions, including balloon dilatation and membranectomy, have emerged as treatment options for CDS.^20,21)^ Although clinical experience with these 2 endoscopic techniques remains limited, several reports have described the use of balloon dilatation even in neonates, indicating that this approach may be feasible regardless of patient age.^20,22)^ While endoscopic interventions may avoid surgery and its associated complications, they require advanced techniques and repeated procedures in some cases. Goring et al. reported that a single endoscopic procedure was successful in 60% of patients with congenital partial duodenal obstruction, whereas the remaining 40% required repeat procedures or conversion to surgery.^23)^ Notably, all cases involving annular pancreas required surgical intervention, suggesting that careful patient selection is essential for endoscopic treatment. In the present case, although endoscopic intervention might have been feasible, we chose to perform an open procedure via an inverted Y-shaped umbilical incision, given that endoscopic management of CDS is not well established, thereby ensuring procedural safety. The surgical outcome was favorable, and the postoperative course was uneventful.

## CONCLUSIONS

This report describes a rare case of CDS that presented late and was triggered by food impaction. Our case highlights the importance of comprehensive noninvasive evaluation in such patients, including CT and contrast imaging, which facilitated both endoscopic removal of the impacted food and definitive surgical treatment of the duodenal stenosis. Clinicians should consider CDS in the differential diagnosis of sudden-onset vomiting in children, maintaining a high index of suspicion even in older patients with trisomy 21.

## DECLARATIONS

### Availability of data and materials

The patient’s data are unavailable because of the need to protect personal information.
